# Long-Term Plasticity in Reflex Excitability Induced by Five Weeks of Arm and Leg Cycling Training after Stroke

**DOI:** 10.3390/brainsci6040054

**Published:** 2016-11-03

**Authors:** Taryn Klarner, Trevor S. Barss, Yao Sun, Chelsea Kaupp, Pamela M. Loadman, E. Paul Zehr

**Affiliations:** 1 Rehabilitation Neuroscience Laboratory, University of Victoria, Victoria, BC V8W 3P1, Canada; tklarner@uvic.ca (T.K.); tsbarss@uvic.ca (T.S.B.); yaosun@uvic.ca (Y.S.); ckaupp@live.ca (C.K.); ploadman@telus.net (P.M.L.); 2 Human Discovery Science, International Collaboration on Repair Discoveries (ICORD), Vancouver, BC V5Z 1M9, Canada; 3 Centre for Biomedical Research, University of Victoria, Victoria, BC V8W 2Y2, Canada; 4 Division of Medical Sciences, University of Victoria, BC V8P 5C2, Canada

**Keywords:** stroke, plasticity, rehabilitation, gait, EMG, reflexes

## Abstract

Neural connections remain partially viable after stroke, and access to these residual connections provides a substrate for training-induced plasticity. The objective of this project was to test if reflex excitability could be modified with arm and leg (A & L) cycling training. Nineteen individuals with chronic stroke (more than six months postlesion) performed 30 min of A & L cycling training three times a week for five weeks. Changes in reflex excitability were inferred from modulation of cutaneous and stretch reflexes. A multiple baseline (three pretests) within-subject control design was used. Plasticity in reflex excitability was determined as an increase in the conditioning effect of arm cycling on soleus stretch reflex amplitude on the more affected side, by the index of modulation, and by the modulation ratio between sides for cutaneous reflexes. In general, A & L cycling training induces plasticity and modifies reflex excitability after stroke.

## 1. Introduction

The arms and the legs are coupled in the human nervous system such that activity in the arms affects activity in the legs and vice versa. In quadrupeds, forelimb–hindlimb coordination is well documented and has been attributed to propriospinal linkages between cervical and lumbosacral spinal central pattern-generating networks [1,2,3,4,5,6]. Bipedal human locomotion is likely built upon elements of quadrupedal coordination [2,5], where it involves coordination of all four limbs. Only indirect evidence for quadrupedal locomotor linkages exists, however.

The modulation of reflex amplitudes can be used to probe for changes in interlimb neural activity [4,7]. Investigations of soleus stretch and H-reflex modulation during rhythmic arm movement provide evidence of neuronal coupling between the arms and the legs [2,3,8,9,10]. Examining cutaneous reflexes during rhythmic movements can also probe for interactions between the limbs. In this context, a widespread interlimb network is revealed by the extensive distribution of reflexes across many muscles in both the arms and the legs regardless of which limb is directly stimulated [4,11,12]. In addition, phase-dependent modulation found in muscles of all four limbs during rhythmic movement is suggestive of coupling between segmental spinal networks [12,13,14,15,16]. Regulation of rhythmic arm and leg movement is supported by somatosensory linkages in the form of interlimb reflexes [12,17,18] and neural coupling between lumbar and cervical spinal cord networks [10,19,20,21,22].

Exploiting the neural and mechanical linkages between the arms and legs, which are inherent parts of human locomotion, could enhance the recovery of walking for those who have suffered neurological damage such as a stroke [2,5,23,24]. Incorporating paradigms for locomotor rehabilitation that include rhythmic arm movements, as with arm and leg (A & L) cycling, may enhance leg activity [2,5,23,25]. Only using the arms for postural and weight-bearing activity (e.g., on parallel bars or handrails), as is commonly applied in traditional locomotor rehabilitation, may actually inhibit rhythmic stepping with the legs [25]. Conversely, when arm activity is facilitated with locomotor-like arm and leg movements in those with incomplete cervical spinal cord injury, leg muscle activity is facilitated [26]. Allowing a normal simultaneous and reciprocating arm action may facilitate stepping and may be an important component needed to harness neural coupling to help improve motor output for the legs during walking [23,27].

A complication of stroke is alterations in interneuronal pathways, stemming from damage to supraspinal centers, which disrupts some of the descending regulation [28,29]. The decreased influence of the corticospinal tract fails to produce the appropriate suppressions associated with normal reflex activity [30]. It is this abnormal neural integration that contributes to reduced walking ability. However, connections between the arms and legs remain partially viable, despite the fact that stroke typically presents with hemiparesis resulting in a more affected (MA) and less affected (LA) side [31,32,33]. Partial preservation of the descending modulatory effects of rhythmic arm cycling on lumbosacral spinal cord excitability can be seen after stroke, where arm cycling modulates the soleus H-reflex [34] and stretch reflex [35]. In addition, the protective stumbling corrective response, ordinarily observed in healthy participants during walking, remains partially intact in stroke participants [32,36]. Altered neural connectivity following stroke produces impairments in limb function with a stereotypical bias of arm flexor and leg extensor activity, resulting in excessive activation coupling between the upper and lower extremities [37].

Although neural pathways are corrupted bilaterally after stroke, residual connectivity in spinal networks provides a substrate for training-induced plasticity arising from A & L cycling training [38]. The extent to which A & L cycling training in stroke could modify plasticity in reflex excitability remains unknown. Thus, the objective of this project was to test if neurophysiological changes in reflex excitability are sensitive to A & L cycling training. We hypothesized that A & L cycling training would improve interlimb reflex excitability as assessed by changes in stretch and cutaneous reflex amplitudes. Recently, we have shown that A & L cycling training successfully improves walking after stroke [39], and results from this study may have implications for the mechanistic understanding of plasticity and training transfer following rehabilitative locomotor training in clinical populations.

## 2. Materials and Methods

### 2.1. Participants

Nineteen individuals with chronic stroke (more than six months postinfarct) were enrolled in the study. To assist with determining a participant’s functional status and the clinical features of this population, clinical assessments were performed by a licensed physical therapist before and after A & L cycling training (see Table 1) (ClinicalTrials.gov: NCT02316405). Muscle tone was measured using the modified Ashworth Scale (5 points) at the ankle and knee for the lower limb [40,41] with a graded rating of spasticity scored from 0 to 4, with 0 being flaccid and 4 being rigid. A measure of the basic motor skills necessary for functional ambulation was derived using the 6-point Functional Ambulation Categories Scale, where a level 1 indicates that a patient is non-ambulatory and a level 6 indicates a patient is fully independent [42]. To measure general physical impairment, the Chedoke–McMaster Stroke Assessment [43] was used. Impairment at the arm (A), hand (H), leg (L), and foot (F) were determined using the 7-point activity scale, where score of 1 represents complete independence and a score of 7 represents total assistance. Using the 5-piece Semmes–Weinstein kit of calibrated monofilaments (Sammons Preston Roylan, Cedarburg, WI, USA), ability to discern light touch and pressure was measured in the more affected hand and foot [44]. Reflexes obtained using a reflex hammer were graded on a 0 to 4+ scale, where 0 means a reflex is absent and 4+ represents a hyperactive reflex with clonus for knee jerk (L1) and ankle plantarflexion jerk (S1) [45]. Table 1 outlines participant demographic information and the clinical features of the population as assessed above taken before and after A & L cycling training. Informed written consent was obtained for a protocol (protocol code: 07-480-04d: modulation of reflex function during rhythmic movement and resulting from training interventions) approved by the University of Victoria Human Research Ethics Committee and performed according to the Declaration of Helsinki. 

### 2.2. A & L Cycling Training

**Figure 1:**
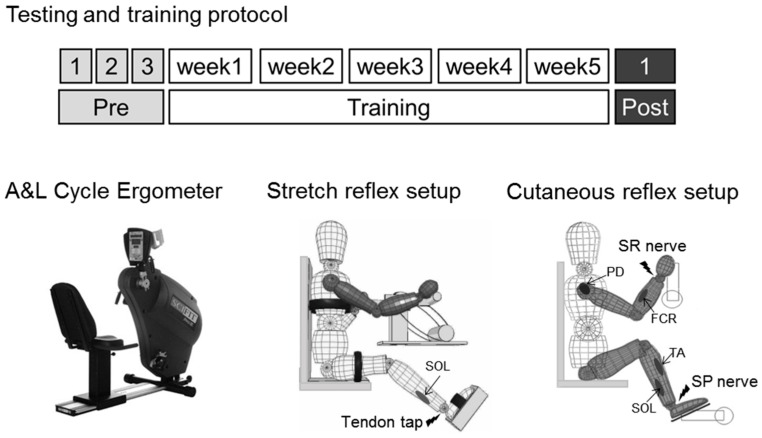
Illustration of the testing and training protocols. A multiple baseline within-subject control design was used for this study. An A & L cycle ergometer (Sci-Fit Pro 2) was used for training. The setups for stretch reflex and cutaneous reflex testing are shown. Muscles of interest are shown with a gray oval, and electrical stimulation is shown with a black lightning bolt. For the stretch reflex setup, a brief vibration was delivered to the triceps surae tendon and the reflex was recorded from the soleus (SOL) muscle, separately for each side. For the cutaneous reflex setup, simultaneous electrical stimulation was applied to the superficial radial (SR) and the superficial peroneal (SP) nerves, and reflexes were recorded bilaterally from the soleus (SOL), tibialis anterior (TA), flexor carpi radialis (FCR), and the posterior deltoid (PD) muscles.

Participants performed A & L cycling training (Sci-Fit Pro 2 ergometer, see Figure 1) three times a week, with 30 min of aggregate activity time per session, for a total duration of five weeks. Most participants completed training on Monday, Wednesday, and Friday. For training, an arm and leg cycling ergometer with coupled upper and lower cranks was used. Linked motion of the cranks for the arms and legs enabled assistance for the weaker limbs where, regardless of deficit, all limbs could be moving, allowing for interlimb coordination during training. It is unknown, however, the contribution from the arms versus the legs since force exerted at each limb was not measured. Mechanical modifications were made to the cycle ergometer to ensure a customized and comfortable fit for each training session. The cranks of the arm and leg ergometer were individually adjusted to the range of motion for each limb of each stroke participant, and hand braces were worn as needed to ensure grip on the handle with the MA hand. During each session, participants were allowed to take short breaks during training, but the aggregate time for each session was always met. In fact, few participants took breaks, and those that did only required them in the early days of training. Participants were expected to tolerate the protocol very well, as this was a modification of a previous protocol where chronic stroke participants performed four trials of 6 min bouts (totaling 24 min) of active A & L cycling [46].

The progressive element of this steady-state training included increasing the resistance of the ergometer over the five weeks in order to maintain the same relative rating of perceived exertion (RPE) score. This is in line with many other post-stroke treadmill training protocols where training volume was increased [47]. Participants were encouraged to exercise at a level sufficient to report an RPE value between 3 and 5, corresponding to a target heart rate (HR) between 50% and 70% of their maximum HR [48]. If a participant reported being on beta blockers, adjustments to target heart rate goals were made [49]. During the training and testing time, participants were also encouraged to maintain their normal activity levels, but not participate in additional research programs or interventions.

All exercise sessions were supervised by a CSEP (Canadian Society for Exercise Physiology) certified exercise physiologist and several laboratory assistants to ensure appropriate monitoring. Exercise sessions were not initiated if a participant’s blood pressure (BP), measured with a digital blood pressure cuff placed over the LA arm, exceeded 140/90 mmHg, in accordance with Canada’s Physical Activity Guidelines [50]. This only occurred, however, for one participant for one exercise session. Exercise was terminated if HR exceeded 85% of the age-predicted maximum, if BP exceeded 200/110 mmHg, or if the participant felt dizzy, nervous, or pains in the chest. No exercise session, for any participant, was stopped due to any of these contraindications. Upon completion of the 30 min in each training session, participants were given 3–5 min to cool down, and remained in the laboratory until BP returned to pre-exercise values.

### 2.3. Multiple Baseline and Post-Test Measures

A multiple baseline within-subject control design was used for this study [51,52]. Figure 1 illustrates the testing and training protocol. Multiple baseline measurements were obtained from participants in three baseline experimental sessions over a period of three to four weeks, with at least six days between baseline sessions. The post-test following training was performed within three days of the last exercise session. At these sessions, the same tests were performed in the same order and environmental conditions (i.e., temperature, noise, lighting, participant position) and session time of day were kept as consistent as possible [53,54,55]. This design allowed for the creation of a reliable and consistent pretest measure that allowed for inspection of spontaneous recovery effects, and provided baseline data against which changes were evaluated. These measures have been previously shown to have high reliability across multiple baseline points [52].

### 2.4. Stretch Reflexes

Soleus stretch reflexes were evoked using an electrodynamic shaker (ET-1126B; Labworks Inc., Costa Mesa, CA, USA), placed over the triceps surae tendons of the LA and MA legs, in separate trials as described previously [35,56]. Constant pressure was applied as best as possible against the tendon, and the shaker was programmed to deliver a single sinusoidal pulse at a frequency of 100 Hz (10 ms duration). A total of 20 pulses were delivered pseudo-randomly with an interstimulus interval between 3 and 5 s. Figure 1 illustrates the stretch reflex setup.

Stretch reflexes were collected under two conditions: (1) with the arms and legs not moving (static) and (2) with the arms cycling rhythmically at 1 Hz (conditioned) while the legs remained static. Arm cycling frequency was set to 1 Hz and participants maintained cycling frequency with the use of visual feedback. All reflexes were evoked at the “7 o’clock” position for the LA hand for both the static and conditioned trials. This position was previously shown to have the largest modulatory effect on H-reflex amplitude [57]. The same procedure was then repeated for the contralateral leg after repositioning the shaker. Each procedure lasted about 2 min, and rest periods of 5 min between them were allowed. Background electromyographic (EMG) activity in the ipsilateral tibialis anterior (TA) and contralateral soleus (SOL) and TA, were also monitored. In both conditions, SOL stretch reflexes were recorded while the participant was relaxed and instructed not to generate any muscle activity.

As a proxy for the intensity of the pulse between conditions, an accelerometer (ADXL193; Analog Devices, Norwood, MA, USA), mounted to the tip of the shaker, recorded stimulation amplitude, as in previous studies [35,58,59]. The peak-to-peak value from the accelerometer signal was obtained based upon the sinusoidal displacement of the shaker tip. Using a standard equation for peak sinusoidal motion, displacement was calculated as:

(1) $$D=\frac{GA}{2\pi^{2}F^{2}}$$

where “*D*” is the peak-to-peak displacement of the tip of the shaker in contact with the tendon, “*G*” is a constant (the acceleration due to gravity), “*A*” is the acceleration measured by the accelerometer in units of gravity, and “*F*” is the frequency of the sinusoid (100 Hz).

Modulation of stretch reflexes due to arm cycling was evaluated by calculating the index of modulation as the change in stretch reflex peak-to-peak amplitude between the static and conditioned trials and then expressed as a percentage (Modulation = [(StretchReflex_ArmCycle_ − StretchReflex_Static_)/StretchReflex_Static_] × 100). Negative values indicate a decrease in reflex amplitude during the arm cycling conditioned trial compared to the static trial. To compare modulation between the LA and MA sides, the difference in stretch reflex amplitudes was calculated by subtracting values for arm cycling from static control amplitudes. A negative value indicates a greater degree of modulation on the LA side and a positive value indicates a greater degree of modulation on the MA side. Background EMG (bEMG) was assessed to monitor possible effects of heteronymous and contralateral muscle activity on reflex amplitudes. For the contralateral SOL and TA bilaterally, bEMG was calculated as the average value of background activity from a 20 ms prestimulus period. Data were normalized to the peak EMG recorded during A & L cycling for each muscle for each session.

### 2.5. Cutaneous Reflexes

The pattern of cutaneous reflex modulation involving combined simultaneous superficial radial (SR) and superficial peroneal (SP) nerve stimulation during A & L cycling was used to assess neurophysiological changes in reflex excitability arising from locomotor training. Cutaneous reflexes were evoked via simultaneous combined surface stimulation of the nerves innervating the dorsum of the hand (SR) and foot (SP) [60]. Electrodes for SR and SP nerve stimulation were placed just proximal to the radial head and on the crease of the ankle, respectively, on the LA limbs. Similar to previous studies [21,32,33,61,62] a Grass S88 stimulator with SIU5 stimulus isolation and a CCU1 constant current unit (Astro-Med Grass Instrument, West Warwick, RI, USA) were used to deliver stimulation in trains of 5 × 1.0 ms pulses at 300 Hz (P511 Astro-Med Grass Instrument). Perceptual and radiating thresholds (RT) were determined as the point at which nerve stimulation produces a perceptible stimulation and the point at which a stimulation produced radiating paresthesia in the entire cutaneous receptive field of that nerve, respectively. Non-noxious intensities were found for each participant and stimulation intensities for the SR nerve were set to 2.2 ± 0.1, 2.0 ± 0.1, 2.3 ± 0.2, and 2.1 ± 0.1 × RT for pretest 1, 2, 3 and the post test, respectively and for the SP nerve stimulation intensities were set to 1.9 ± 0.2, 2.1 ± 0.1, 2 ± 0.1, and 2 ± 0.1 × RT for pretest 1, 2, 3 and the post test, respectively. No significant differences were found in stimulation intensity across test sessions.

EMG data from the soleus (SOL), tibialis anterior (TA), flexor carpi radialis (FCR), and posterior deltoid (PD), from the LA and MA limbs, were collected with surface electrodes placed in bipolar configuration over the muscle bellies of interest. Muscles from all four limbs bilaterally that have been previously associated with interlimb reflex effects were chosen [19,32,33,61,62]. Electrodes were placed on the skin, oriented longitudinally along the fiber direction, in accordance with SENIAM procedures [63]. Electrodes on the upper and lower limbs were placed in the same position at each testing session. This was accomplished by recording cathode and anode electrode distances from anatomical landmarks, using pictures taken at the first session, and by placement of the electrodes by the same experimenter each time. EMG signals were preamplified (×5000), band-pass filtered (100–300 Hz), converted to a digital signal (GRASS P511, AstroMed, West Warwick, RI, USA), and sampled at 1000 Hz using custom-built continuous acquisition software (LabVIEW, National Instruments, Austin, TX, USA). Using custom-written software programs (Matlab, The Mathworks, Inc., Natick, MA, USA) EMG data were full-wave rectified and low-pass filtered at 100 Hz using a fourth-order Butterworth filter.

Participants performed A & L cycling on the same cycle ergometer used for training. Figure 1 illustrates the cutaneous reflex setup. After establishing a consistent steady pace for A & L cycling (55.2 ± 9.2 rpm), data were collected over a 4–6 min trial providing approximately 160 stimulations delivered pseudo-randomly with an interstimulus interval of 1–5 s. Continuous data for A & L cycling were broken into movement cycles with the vertical position of the LA arm indicating the start and end of a cycle. For comparisons between participants, cycle time was normalized to 100%. 

To investigate phase-dependent modulation within each movement cycle, data were broken apart into 8 equidistant phases. Phases 1–4 represent the arm and leg power phase, corresponding to the LA arm at top dead center (0 deg) to full extension of the arm and leg (180 deg) [19]. Evoked reflexes in all muscles tested were aligned to delivery and averaged together. The stimulus artefact was removed from the reflex trace and data were then low-pass filtered at 30 Hz using a dual-pass, fourth-order Butterworth filter. For reflexes within each phase, the average trace from the non-stimulated data was subtracted from the stimulated average trace to produce a subtracted EMG reflex trace. Cutaneous reflexes were quantified as the average cumulative reflex over 150 ms following stimulation within each of the 8 phases [64,65]. A positive value indicates overall facilitation while a negative value (only revealed with background activity) indicates overall inhibition [19,66,67]. This process reveals the general trend in evoked responses in the muscles tested [68]. However, it does reduce the ability to identify reflex reversals, as this method mixes facilitations and suppressions losing some of the temporal and spatial characteristics of the response [66,69]. Background EMG (bEMG) levels were investigated for a corresponding comparison of reflex amplitudes between tests. A modulation index for change in bEMG across phases for each muscle was calculated (Modulation = [(bEMG_max_ − bEMG_min_)/bEMG_max_] × 100) and this measure provides an index of overall amplitude modulation independent of the pattern of modulation across A & L cycling phases [32]. Cutaneous reflex amplitudes and background EMG for each subject were normalized to the peak value of the unstimulated EMG for that muscle across the movement cycle for A & L cycling. A modulation index for change in reflexes across phases for each muscle was calculated (Modulation = [(Reflex_max_ − Reflex_min_)/bEMG_max_] × 100). The ratio between the LA and MA modulation index for each muscle was also determined.

### 2.6. Statistics

Using commercially available software (SPSS 18.0, Chicago, IL, USA) pretest and post-test data were compared with two different methods; a within-subjects and a between-subjects analysis to evaluate the extent to which arm and leg cycling training altered reflex modulation. Two statistical methods are provided as a means of increasing statistical testing procedures for multiple baseline designs. 

For the within-subject analyses, post-test data were compared to the 95% confidence interval (CI) created from three pretest sessions. To establish the 95% CI for each measure, variability was computed from three pretest sessions and used to create a data range with which the post-test value was compared. If the post-test value fell outside the 95% CI range, it was considered statistically significant for that participant [70]. A graph illustrating the 95% CI across pretests sessions and the post-test value is included for each measure. 

For the between-subject analysis, using group data, we used repeated-measures ANOVA to evaluate the extent to which arm and leg cycling training altered reflex modulation. For stretch reflex and modulation index parameters, a 1 × 4 (time; test sessions) repeated-measures ANOVA was used. For cutaneous reflex parameters an 8 (phase) × 4 (time; test sessions) repeated-measure ANOVA was used and only main effects and interaction effects for time are reported and considered. For comparison between baseline and post-test data, a repeated-measures ANOVA was first performed in a planned contrast to examine differences across the three pretest sessions. If no difference was found, data were pooled together to create an average pretest value, which was compared to the post-test. Assumptions for ANOVA were evaluated for parametric tests for a within-subject design with statistical significance set at *p* ≤ 0.05. As an additional measure of the magnitude of any differences between pre–post, the observed effect size for post-test differences for stretch and cutaneous reflex parameters is also reported using Cohen’s *d*. In this calculation we used the conventional small effect as *d* = 0.2, a medium effect as *d* = 0.5, and a large effect as *d* = 0.8 [71].

## 3. Results

### 3.1. NOTE

Table 2 summarizes results from the single-participant statistical tests that are discussed below. The number of participants with a significant post-test value is reported for each variable in the table. A graph illustrating the average (middle gray circle) and 95% CI across three pretests sessions (upper and lower outside gray circles) and the post-test value (black circle) is included for each measure. For these graphs, post-test variables that fall outside of the 95% CI are of interest and the actual values can be found on the subsequent graphs for each variable. For stretch reflex modulation for the MA SOL and for the ratio of stretch reflex modulation between the LA and MA sides, the average post-test value fell outside of the 95% CI from the pretest sessions, indicating a significant training effect. For cutaneous reflexes, the modulation index for the MA FCR and the modulation index ratio for the SOL and TA have a post-test value that fell outside of the 95% CI band from the pre-test sessions. For bEMG, the modulation index for the MA TA, MA FCR and LA PD, and modulation index ratio for the TA, the average post-test value fell outside of the 95% CI from the pretest sessions. In the text below, the percent change in each significant measure, and the number of participants showing a significant post-test effect, is reported.

### 3.2. Stretch Reflexes

**Figure 2:**
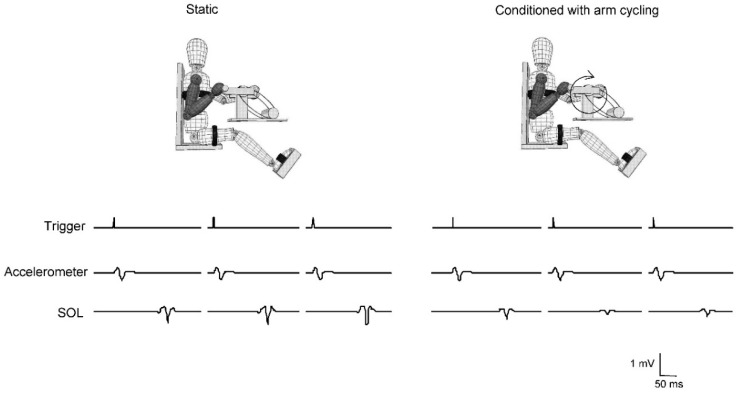
Representative traces in static and conditioned trials. Trigger, accelerometer, and soleus stretch reflex amplitudes from a single subject showing suppression of reflex amplitude in the conditioned arm cycling trial.

Figure 2 shows example representative traces for the shaker trigger, the accelerometer input, and for the soleus stretch reflex for the static and conditioned trial from a representative participant. Suppression due to arm cycling can be seen for the soleus stretch reflex amplitude with a constant trigger input.

No significant differences for input trigger amplitude between the static and conditioned trials for the pretests and the post-test were found. A constant displacement of the pulse was maintained during all stretch reflex trials within each experimental session. For the LA side, the shaker displacement was 0.51 ± 0.23, 0.52 ± 0.15, 0.47 ± 0.13, and 0.52 ± 0.12 mm for pre1, pre2, pre3, and post-tests, respectively. For the MA side the shaker displacement was 0.48 ± 0.17, 0.49 ± 0.11, 0.44 ± 0.13, and 0.48 ± 0.16 mm for pre1, pre2, pre3, and post-tests, respectively. There was no significant difference in shaker displacement between the LA and MA sides. Therefore, differences in stretch reflex amplitude were not caused by changes in input size and modulation due to arm cycling. 

**Figure 3:**
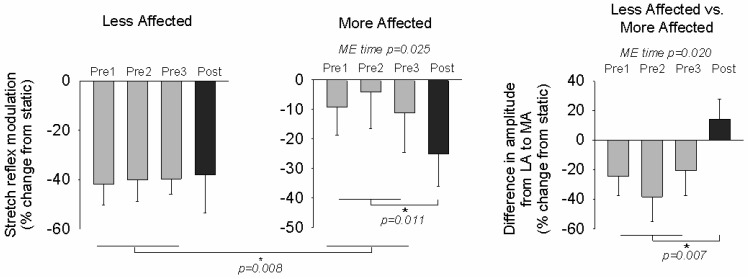
Modulation of stretch reflex by arm cycling. Bar graphs are means (± SEM) averaged across all participants for baseline and post-test values. Repeated-measures ANOVA was used to assess the effects of A & L cycling training. Main effects (ME) are listed for reflexes. For comparison between baseline average and post-test data, *p*-values are shown for each test. * indicates significant differences between the pre-test average and the post-test value. For the LA side, MA side, and difference in amplitude modulation between the LA and MA sides.

Figure 3 shows the percent change in the conditioned soleus stretch during arm cycling compared to static control for the baseline and post-tests, and the difference in amplitude modulation between the LA and MA sides averaged across all participants. Soleus stretch reflex modulation on the LA side increased by 19.9% in 10 of the 19 participants from the within-subject analysis, but there was no significant main effect of time or difference in the pretest average and post-test value across participants. For all tests, the LA a significant main effect of time (F_(3,54)_ = 3.497, *p* = 0.025, *d* = 0.733). When comparing the baseline soleus stretch reflex was reduced with arm cycling compared to static. For the MA SOL, there was average with the post-test, the MA SOL shows an 80.8% increase in the inhibitory effect of arm cycling on soleus stretch reflex excitability in 15 of the 19 participants (F_(1,18)_ = 8.983, *p* = 0.011, *d* = 1.034) after training.

At the pretest sessions there was a significantly larger degree of modulation on the LA side compared to the MA side (F_(1,18)_ = 9.558, *p* = 0.008, *d* = 1.012), where pretest stretch reflex amplitudes for all participants were reduced with arm cycling by 38.4% for the LA side compared to 10.9% for the MA side. After training, the post-test revealed there was no longer a significant difference between the sides in stretch reflex inhibitory modulation, where now both LA and MA stretch reflexes for all participants are reduced by 36.4%. When comparing the absolute difference in the degree of modulation between the LA and MA sides after training, there was a significant main effect of time across all sessions (F_(3,54)_ = 3.770, *p* = 0.020, *d* = 0.763). With the baseline average and post-test values there was a significant effect (F_(1,18)_ = 10.190, *p* = 0.007, *d* = 0.839) such that the relative effect of modulation during arm cycling for the pretests was 27.8% stronger for the LA side and for the post-test 14.2% stronger on the MA side in 10 of the 19 participants.

**Figure 4:**
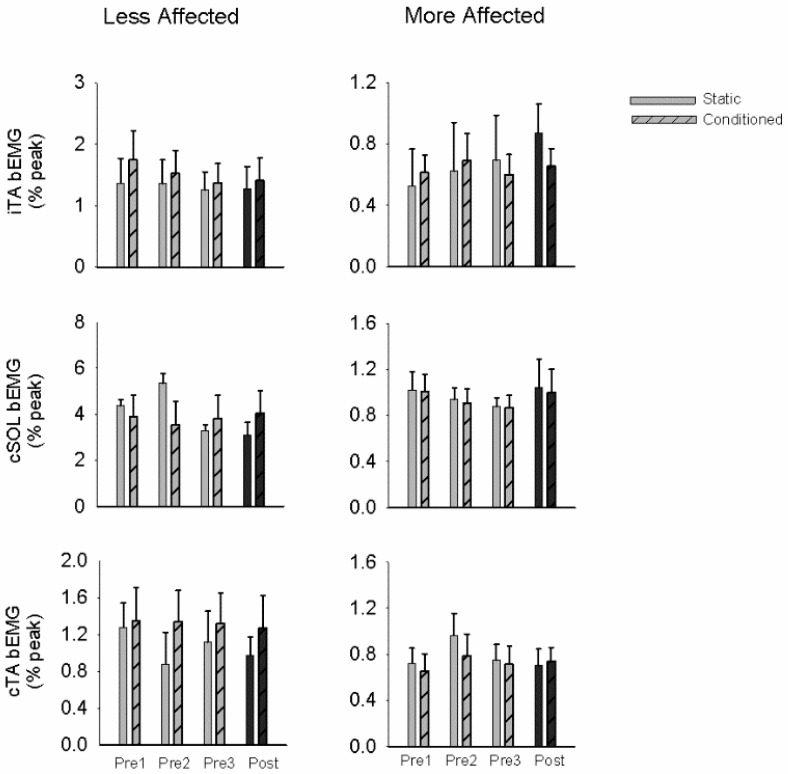
Background EMG during stretch reflex testing. Bar graphs are means (± SEM) averaged across all participants for static and conditioned stretch reflexes on the less affected and more affected sides. Background EMG values are reported in mV. For ipsilateral (i) TA and contralateral (c) SOL and TA. Repeated-measures ANOVA was used to assess the effects of A & L cycling training with significance set at *p* ≤ 0.05. No significant effects where found.

Figure 4 shows background EMG activity during testing of soleus stretch reflex amplitude modulation by arm cycling in the LA and MA sides. No significant differences were found for background EMG between the static and conditioned trials for the pretests or the post-test.

### 3.3. Cutaneous Reflexes

**Figure 5:**
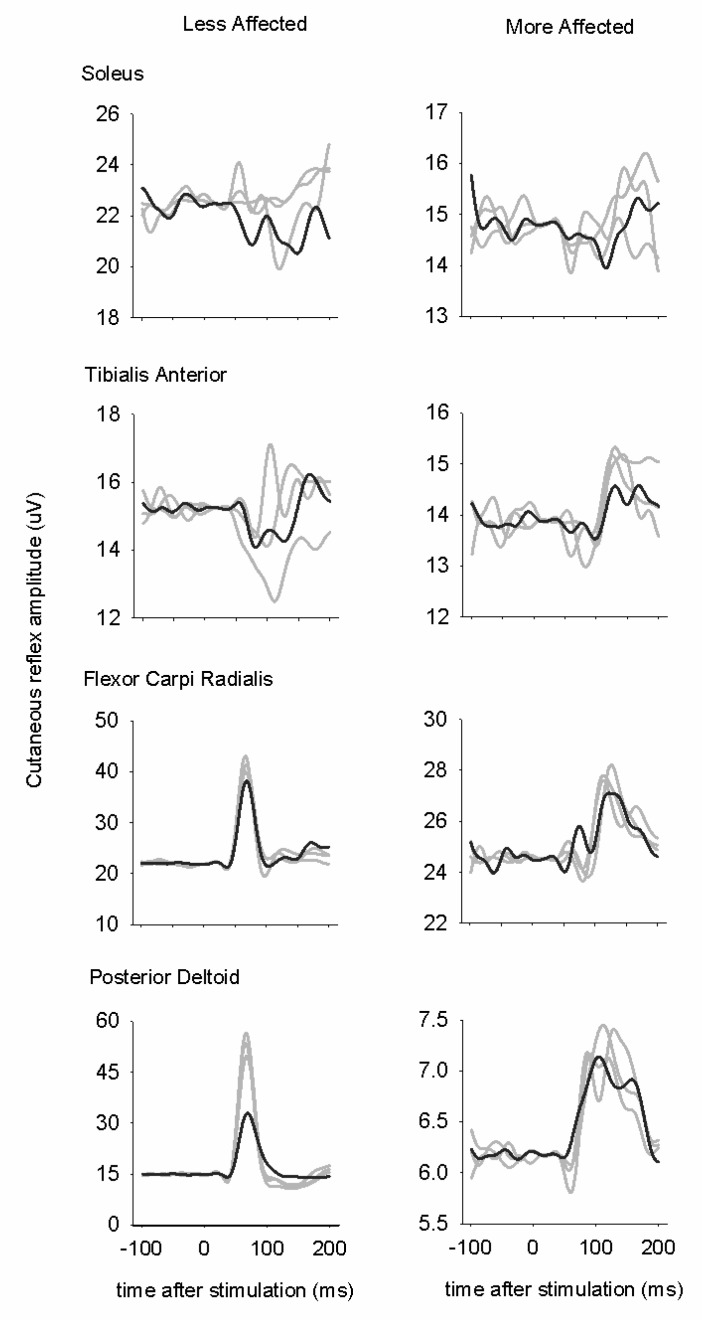
Grand average cutaneous reflex traces for all phases for A & L cycling. Line graphs are averages across all participants for three baseline tests (light gray lines) and for the post-test (dark gray line). The stimulus artefact beginning at time 0 has been blanked out and replaced by a flat line. Stimulation was applied to the superficial radial nerve of the hand and the superficial peroneal nerve of the foot on the LA side.

Figure 5 shows grand average data across all phases for all participants for reflexes evoked during A & L cycling. This representation is useful for showing the general trends in the evoked responses independent of the phase of the step cycle [60,68]. Such grand averages taken irrespective of phase only provide an “at a glance” qualitative assessment of changes, and provide a visual representation of the strongest effects. However, features such as phase-dependent reflex reversals, which occur only in a few phases, actually are obscured by this process and, in fact, may appear as “noise”. For that reason, error bars have also been omitted. Cutaneous reflexes are observed in all muscles tested bilaterally from LA hand and foot stimulation.

**Figure 6:**
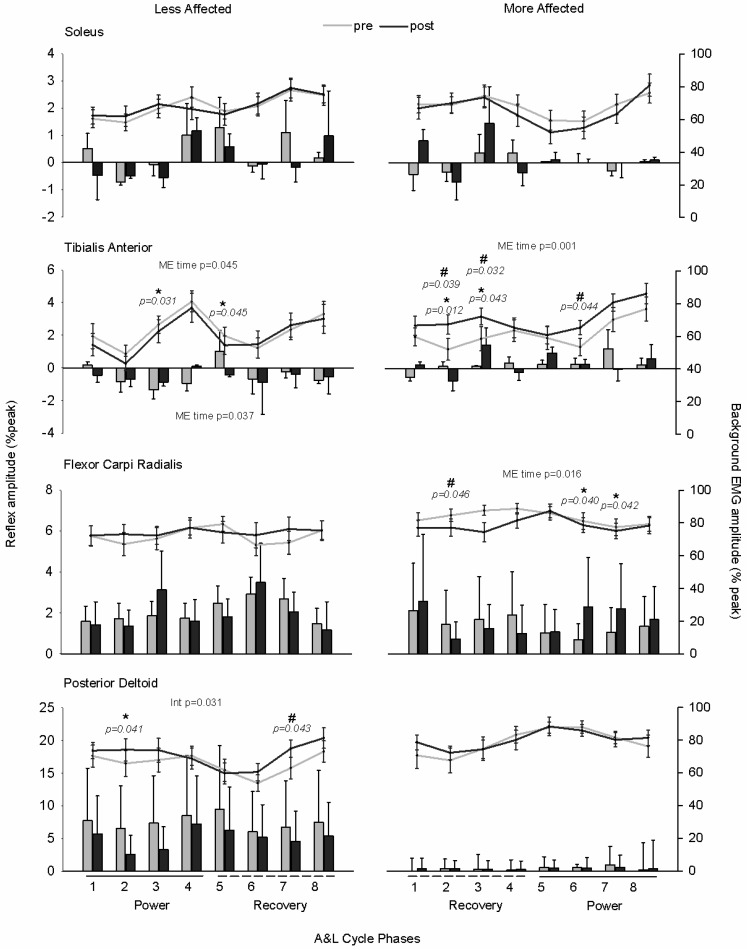
Normalized background EMG and reflex amplitudes during A & L cycling. Background EMG is shown in line plots and reflex amplitude is shown in bar plots. Values are means (± standard error) averaged across all participants and normalized to the peak undisturbed EMG during A & L cycling. The horizontal bars below the *y*-axes represent the power (solid line) and recovery (dotted line) phase of A & L cycling. Phases one to four represent the arm and leg power phase, corresponding to the LA arm at top dead center (0 deg) with full extension of the arm and leg (180 deg). Repeated-measures ANOVA was used to assess the effects of A & L cycling training. Main effects (ME) and interactions (Int) are listed for reflexes above each subplot and ME are listed for bEMG below each panel. For comparison between baseline average and post-test data, *p*-values are shown for each test. Significant differences between the pretest average and the post-test value are indicated with # for background EMG and * for reflex amplitude.

Figure 6 shows normalized background EMG (lines) and reflex amplitudes (bars) during A & L cycling. The horizontal bars below the *y*-axes represent the power (solid line) and recovery (dotted line) phases of A & L cycling. As there were no significant differences between pretest data samples, for simplification the average value across the three tests is shown.

The results of omnibus ANOVA for all phases and tests reveal main effects of time and phase. As main effects of phase are not of interest, we only addressed main effects of time. Results revealed that there were main effects of time for the MA TA (F_(3,54)_ = 3.947, *p* = 0.001, *d* = 0.976), LA TA (F_(3,54)_ = 2.720, *p* = 0.045, *d* = 0.768), and MA FCR (F_(3,54)_ = 2.620, *p* = 0.016, *d* = 0.873). For the LA PD there was a significant interaction for phase and time (F_(21,273)_ = 1.699, *p* = 0.031, *d* = 0.966). When looking at specific phases for reflex amplitudes, there are significant differences between the baseline average and post-test values for muscles with significant main effects. For the MA TA, significant effects were found for phase 2 and 3 (F_(1,18)_ = 8.561, *p* = 0.012, *d* = 0.435 and F_(1,18)_ = 5.049, *p* = 0.043, *d* = 0.400, respectively). For the LA TA, significant effects were found for phase 3 and 5 (F_(1,18)_ = 5.813, *p* = 0.031, *d* = 0.254 and F_(1,18)_ = 4.486, *p* = 0.045, *d* = 0.362, respectively). For the MA FCR, there were significant differences found when comparing the baseline average and post-test values for phase 6 (F_(1,18)_ = 5.094, *p* = 0.040, *d* = 0.204) and 7 (F_(1,18)_ = 4.601, *p* = 0.042, *d* = 0.215). For the LA PD, significant effects were found for phase 2 (F_(1,18)_ = 3.561, *p* = 0.041, *d* = 0.379). In summary, there were significant main effects of training on cutaneous reflexes at different phases for the MA and LA TA, MA FCR, and LA PD. 

Investigating background EMG levels between conditions allows for comparison of reflex amplitudes that cannot be explained by simple gain scaling with motoneuronal pool excitability. The results of omnibus ANOVA for all phases and test sessions reveal main effects of time for background EMG in the LA TA (F_(3,54)_ = 3.149, *p* = 0.037, *d* = 0.682). When comparing the pretest average to post-test values for bEMG at specific phases of A & L cycling, there were significant differences between baseline and post-test values for several muscles. For MA TA, A & L cycling training increased activity for phase 2 (F_(1,18)_ = 5.373, *p* = 0.039, *d* = 0.555), 3 (F_(1,18)_ = 5.871, *p* = 0.032, *d* = 0.494), and 6 (F_(1,18)_ = 5.044, *p* = 0.044, *d* = 0.564). For the MA FCR, bEMG was decreased for phase 2 following training (F_(1,18)_ = 5.956, *p* = 0.046, *d* = 0.371) and was increased for the LA PD bEMG for phase 7 following training (F_(1,18)_ = 5.063, *p* = 0.043, *d* = 0.464). In summary, there were significant main effects of training on background EMG at different phases for the MA TA and FCR, and LA PD.

**Figure 7:**
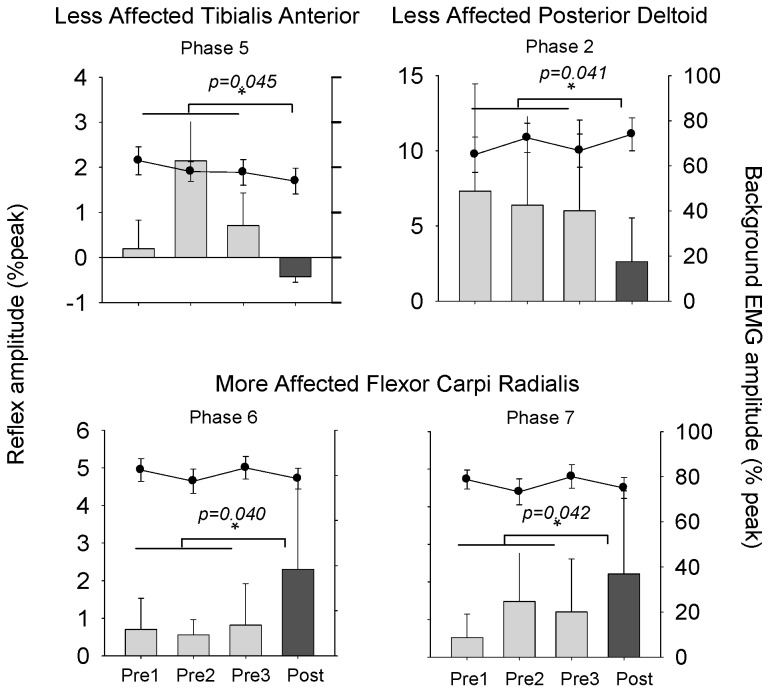
Normalized background EMG and reflex amplitudes at specific phase of interest during A & L cycling. Bar graphs are means (± standard error) for reflex amplitude averaged across all participants for baseline and post-test values. Line graphs are means (± standard error) for bEMG. Repeated-measures ANOVA was used to assess the effects of A & L cycling training with significance set at *p* ≤ 0.05. *p*-values are shown for each test. * indicates significant differences between the pretest average and the post-test value. There were no significant differences for bEMG.

Figure 7 shows specific phases of interest for which there were significant effects of training on reflex amplitude. Phase 5 is shown for the LA TA and phase 2 is shown for the LA PD muscle. For the MA FCR, phases 6 and 7 are shown. For bEMG data in Figure 7, there were no significant effects of training on modulation of these values.

### 3.4. Modulation Index

**Figure 8:**
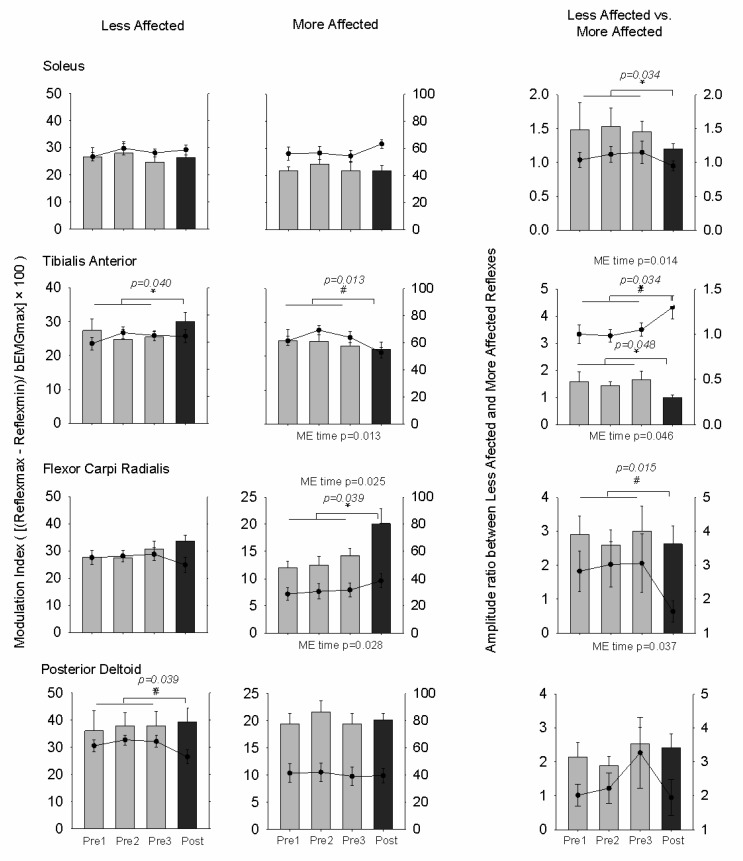
Modulation indices and amplitude ratios for reflexes for all muscles during A & L cycling. Bar graphs are means (± standard error) averaged across all participants for baseline and post-test values. Line graphs are means (± standard error) for bEMG. Repeated-measures ANOVA was used to assess the effects of A & L cycling training with significance set at *p* < 0.05. Main effects (ME) are listed for reflexes above each subplot and ME are listed for bEMG below each panel. For comparison between baseline average and post-test data, *p*-values are shown for each test. * For cutaneous reflex and # for bEMG indicates significant differences between the pretest average and the post-test value.

Figure 8 shows the modulation index for the muscles on the LA and MA side and the ratio between LA and MA modulation for cutaneous reflexes and bEMG. Results from omnibus ANOVA reveal that there was a significant main effect of time for the MA FCR (F_(3,54)_ = 3.809, *p* = 0.025, *d* = 0.631). When comparing the pretest average to the post-test, we found that modulation increased by 30.3% in 11 out of 19 participants for the MA FCR (F_(1,18)_ = 5.026, *p* = 0.039, *d* = 0.771) following training. Although no significant main effect was found, we saw a 9.8% increase in 8 out of 19 participants (F_(1,18)_ = 4.999, *p* = 0.040, *d* = 0.359) for the LA TA when comparing with the pretest average. There was a significant main effect of time for the ratio of modulation for the TA (F_(3,54)_ = 3.889, *p* = 0.014, *d* = 0.631). Following training, modulation was decreased by 54.6% in 10 out of 19 participants (F_(1,18)_ = 5.345, *p* = 0.034, *d* = 0.613). When comparing the pretest average to the post-test we found that modulation was also decreased by 14.2% in 12 out of 19 participants for the SOL (F_(1,18)_ = 4.566, *p* = 0.048, *d* = 0.317). In summary, the modulation index for cutaneous reflexes was changed after training for the MA FCR and LA TA and the amplitude ratio between LA and MA reflexes was changed for the SOL and TA. 

For bEMG, there were significant main effects of time for the MA TA (F_(3,54)_ = 4.095, *p* = 0.013, *d* = 0.804), LA PD (F_(3,54)_ = 3.656, *p* = 0.043, *d* = 0.731), and MA FCR (F_(3,54)_ = 3.396, *p* = 0.028, *d* = 0.719). When comparing the pretest average to the post test, we found that modulation decreased by 23.1% in 9 out of 19 participants following training in the MA TA (F_(1,18)_ = 8.372, *p* = 0.013, *d* = 0.732) and decreased by 20.2% in 8 out of 19 participants in the LA PD (F_(1,18)_ = 5.219, *p* = 0.041, *d* = 0.518). For the ratio of modulation for background EMG, there was a significant main effect of time for the TA (F_(3,54)_ = 3.311, *p* = 0.046, *d* = 0.697) and the FCR (F_(3,54)_ = 3.150, *p* = 0.037, *d* = 0.789). When comparing the pretest average to the post test, we found that the ratio increased by 21.2% in 7 out of 19 participants for the TA (F_(1,18)_ = 5.685, *p* = 0.034, *d* = 0.617) and decreased by 20.2% in 10 out of 19 participants for the FCR (F_(1,18)_ = 7.988, *p* = 0.015, *d* = 0.737) following training. In summary, the modulation index for background EMG was changed after training for the MA TA and LA PD, and the modulation index ratio was changed for the TA and FCR.

## 4. Discussion

Here we demonstrate that A & L cycling training can lead to long-term plasticity in reflex excitability after stroke. This plasticity was observed as changes in stretch reflex and cutaneous reflex activity prior to and after five weeks of A & L cycling training. These results reveal that plasticity in spinal networks occurs as part of the adaptations induced by rehabilitative locomotor training for stroke participants. Testing for plasticity in reflex pathways may be used to probe changes in neural connectivity as a result of rehabilitation interventions for patient populations.

### 4.1. Plasticity in Stretch Reflex Modulation

Examining changes in arm and leg coupling effects on soleus stretch reflexes shows plasticity following A & L cycling training. Results from this study showed plasticity in stretch reflex pathways for the MA side producing increased modulatory effects of arm cycling following training. This observation was supported by both the results of a within-participant and repeated-measures analysis. Since heteronymous and contralateral muscle activity influences stretch reflex excitability via the soleus Ia pathway [72,73,74,75], bEMG activity was recorded to monitor possible effects on the soleus stretch amplitude. The contralateral SOL and ipsilateral and contralateral TA muscles showed no statistically significant differences between the static and conditioned trials. Therefore, the bEMG activity of the above muscles cannot be implicated as a source of the significant suppression seen on the MA stretch reflex amplitude with arm cycling.

A & L cycling training could have altered Group Ia presynaptic inhibition (PSI) to cause the difference in modulatory effects of arm cycling on MA SOL stretch reflex excitability seen after training here. Modulation of Ia PSI by rhythmic activity in cervical spinal cord oscillators is an important mechanism for reflex modulation in the legs in response to arm cycling [3,22]. This mechanism appears partially preserved after stroke [34,35], although participants with stroke have difficulty in modulating the excitability of inhibitory pathways in different motor tasks [76,77,78]. However, given the involvement of the fusimotor system on excitability in the stretch reflex pathway [79,80], changes in gamma bias related to changes in supraspinal regulation could also have contributed to changes in MA SOL stretch reflex excitability. Another possible source of the improved reflex modulation is that sensory function was strengthened and restored after training, allowing for better transmission of afferent signals.

The A & L cycling training produced a change in modulation of stretch reflex amplitude for the LA and MA sides. The amount of suppression on the MA side was restored to the relative levels seen on the LA side, and showed an increased depth of modulation after A & L cycling training. Initial differences between sides in stretch reflex modulation during arm cycling could be due to asymmetrical differences of presynaptic regulation following stroke. Arm and leg muscles show reduced Ia PSI [81] and only higher threshold motor units can be modulated on the MA side [34]. Therefore, there is a larger potential for plastic modulation on the MA side compared to the less impaired LA side.

### 4.2. Plasticity in Cutaneous Reflex Modulation

Responses to cutaneous stimulation showed altered modulation patterns following training. When examining specific functional phases for A & L cycling, reflexes in the LA SOL, LA TA, MA TA, MA FCR, and LA PD are differentially modulated following A & L cycling training. There were also some instances where there were differences in background EMG as a result of training, which could contribute to changes in reflex activity—see, for example, MA TA. Additionally, in a few cases the background changes were in line with changes in reflex activity in the LA TA, MA FCR, and LA PD, for example. Thus, for these muscles, only phases where there were no concomitant changes in bEMG were considered further. 

In some cases, the modulation following training represents a return to what one “normally” observes in these networks. For example, cutaneous reflex activity in the LA TA is mainly suppressive during locomotion after stroke [36] or hereditary spastic paraparesis [30]. Here, in the LA TA, modulation switches from excitation, seen during all three pretests, to inhibition at the transition from the power to recover phases following A & L cycling training. In the LA PD, stimulation following training produced decreased facilitation in the power phase, which also represents a “return” to what is typically observed in neurologically intact subjects [32,33]. These results suggest that neurophysiological reflex excitability can be modified by A & L cycling training, and in some cases, can help restore cutaneous reflex transmission.

In the wrist flexor FCR muscle on the MA side, reflexes were facilitated specifically in the power phase, following A & L cycling training, seen with both the within-participant and repeated-measures analysis. This is evidence for increased response to stimulation from the contralateral (LA) side. Increased reflex activity from contralateral stimulation could represent an increase in crossed responses following training, where access is to be best gained by stimulating cutaneous fields in the LA hand [32]. Measuring reflexes in contralateral muscles gives information on crossed responses and can provide an index of the participation of the contralateral side of the body. By using an index of modulation it is also possible to see how the depth of reflex modulation changed with A & L cycling training. In the LA TA and MA FCR, modulation increased, representing an increased depth of modulation following training. When examining the grand average reflex traces from cutaneous stimulation, reflexes on the MA side were lower in amplitude than reflexes on the LA side. This was not surprising, given that stimulation was applied to the LA side. However, after training, both within-participant and repeated-measures analysis revealed that reflexes in the leg muscles had a decreased amplitude ratio, representing a normalization of response amplitudes between the LA and MA sides.

### 4.3. Methodological Considerations

These results should be interpreted in light of several methodological considerations that must be acknowledged. Because of the linked motion of the cranks, it is unknown how much propulsion was powered from the legs versus the arms. It is possible that participants who imparted the most force via the arm-crank were most likely to respond to the intervention. Future research should focus on measuring the relative participation from the arms versus the legs during training, and how this correlates to improvement. Additionally, features of the stroke, including the time since injury, location of the stroke infarct, or degree of impairment, could have affected the likelihood of A & L cycling training inducing plasticity. However, we did not find an association between these characteristics and our outcomes, instead observing some aspects of plasticity induced irrespective of time after injury or location of stroke. For example, even a participant who was 29 years post-stroke showed plastic changes alongside participants who were 2 years post-stroke. Nevertheless, future research on this topic is warranted.

Concomitant changes in bEMG, as a result of the training, could affect both stretch and cutaneous reflex amplitudes between tests. As a result, only trials and phases without changes in bEMG were considered in detail. In addition, evoked responses may vary somewhat from one session to the next, making comparison across multiple tests difficult. However the measures used here have been previously shown to have high reliability across multiple baseline points [52], and for the phases with functionally significant changes, no differences in reflex modulation were seen across several pretest sessions.

Lastly, statistical design for multiple baseline designs is limited, and little consensus exists across the literature on the best statistical design to use. Therefore, we offer two types of statistical assessments: a within-participant design, whereby we use the confidence interval from three pretest sessions to compare the post-test value, and a design using repeated-measures ANOVA to evaluate significant post-test effects with a planned contrast. For many of our outcome variables, results from both statistical designs support the same conclusion, therefore, we are confident in our observations.

### 4.4. Plasticity and Locomotor Rehabilitation

In response to motor training, neural circuits have the plastic ability to alter their structure and function [82,83,84,85]. Plasticity in somatosensory reflex pathways has been well documented in humans, where rapid, short-term, and persistent chronic changes are observed [38]. Improvements in locomotor ability and walking, as a result of chronic adaptation in neural circuits, is the goal of locomotor rehabilitation. Improvements in walking observed following locomotor rehabilitation for those with spinal cord injury and stroke [29,86,87,88,89] could be due to plasticity in neural networks. Locomotor training improves premotoneuronal control, as seen by (1) the reversal of presynaptic facilitation to presynaptic inhibition of soleus monosynaptic motoneurons at rest; (2) phase-dependent modulation of presynaptic inhibition during walking [90,91,92]; and (3) an improvement in soleus H-reflexes modulation, where after 10 days of locomotor training in those with cerebral palsy, reflexes were almost completely suppressed during the swing phase, showing a return to what is normally exhibited by healthy subjects [93]. Plasticity in reflex pathways could reflect an improvement in reflex transmission, leading to more functional reflex modulation and potentially enhancing walking. However, it may not always be beneficial to simply enhance neural coupling, as this may exaggerate abnormal post-stroke effects, but to induce a beneficial adaptation with targeted training aimed at improving function. For example, operant down-conditioning of H-reflexes can be used to control exaggerated responses, leading to enhanced recovery of function and improved walking after incomplete spinal cord injury [94].

Here we have evidence of neural plasticity in the portion of neural linkages that remain accessible after stroke arising from A & L cycling training affecting muscle afferent and cutaneous reflex pathway excitability after stroke. The focus of future research should be to determine exactly how these changes in reflex excitability are associated with the recovery of motor function. We have recently shown that A & L cycling does indeed transfer to improved walking ability after stroke [39] and the changes in reflex plasticity seen here could be related to this observation. The development of new and targeted therapeutic innovations will further the functional improvements for individuals with neurological disorder.

Plasticity within reflex pathways can be used to probe changes in neural connectivity as a result of rehabilitation interventions for patient populations. For example, testing the conditioning effects of arm cycling on the soleus stretch reflex could be used as a proxy for measuring the change in strength of excitability in neural connections between the arms and the legs. Stretch reflex testing, a protocol based on a relatively simple procedure (a tendon tap), might be useful for application in stroke populations, compared to testing the electrically evoked H-reflex pathway, as allodynia is not uncommon in this group. In addition, hyperreflexia, as measured by stretch reflexes, would more clearly reveal reflex modulation associated with the fusimotor system [79]. Testing cutaneous reflex connections in A & L cycling could also be used to evaluate changes in neural coordination. Measuring reflexes in the MA limb, from stimulation of the LA limbs, gives information on crossed responses, and could provide an index of the participation of the contralateral side of the body. Methodologies that could be widely incorporated in the stroke population are of increased use to researchers and clinicians engaged in the conception and refinement of rehabilitative procedures and practice.

## 5. Conclusions

A & L cycling training for stroke participants modifies reflex excitability. This was determined as an increase in the conditioning effect of arm cycling on soleus stretch reflex excitability on the MA side, and as a change in modulation, a change in the index of modulation, and a change in the modulation ratio between the LA and MA sides of cutaneous reflexes. Testing for plasticity in reflex pathways may be used to probe changes in neural connectivity resulting from rehabilitation interventions for clinical populations.

## Figures and Tables

**Table 1:** Summary of participant demographics and results from tests assessing clinical status including a test for muscle tone (modified Ashworth), functional ambulation (FAC), physical impairment (Chedoke–McMaster scale), touch discrimination (monofilament test), and reflex function for stroke participants before and after arm and leg (A & L) cycling training.

*N*	Sex/Age/MA Side/Years Since Stroke	Modified Ashworth (ankle/knee)		FAC (/6)		Chedoke-McMaster (A/H/L/F)		Monofilament (hand/foot)		Reflexes (L1/S1)	
		Pre	Post	Pre	Post	Pre	Post	Pre	Post	Pre	Post
1	M/74/R/5	3/1+	3/1+	4	4	2/2/3/2	2/2/2/3	J4.31/J4.31	J4.31/J4.31	3+/1+	3+/1+
2	F/70/R/2	0/0	0/0	5	5	7/5/7/7	7/7/7/7	J4.31/J4.31	J4.31/J4.31	2+/2+	2+/2+
3	F/45/R/7	1/0	1/0	5	5	5/5/6/5	5/5/6/4	F3.61/J4.31	D2.83/F3.61	0/0	1+/1+
4	M/59/R/3	2/0	2/0	5	5	2/2/4/2	2/2/4/2	T6.65/J4.31	K4.56/J4.31	3+/3+	3+/3+
5	M/82/R/3	0/1	0/1	3	3	4/6/6/5	5/6/6/5	UTF/UTF	UTF/UTF	3+/0	3+/0
6	M/86/L/4	1+/0	1+/0	5	5	7/7/6/5	7/7/6/6	J4.31/T6.65	J4.31/T6.65	0/0	0/0
7	F/80/R/6	0/0	0/0	5	5	3/5/5/5	3/5/5/6	J4.31/J4.31	F3.61/J4.31	0/0	0/0
8	M/59/R/11	1/1	2/1	5	5	5/5/5/4	5/6/6/4	T6.65/T6.65	T6.65/T6.65	3+/4+	3+/3+
9	M/74/R/6	1/0	1/1	5	5	6/5/6/5	7/7/6/5	J4.31/F3.61	F3.61/D2.83	3+/2+	3+/2+
10	M/47/L/6	4/2	2/2	4	4	2/1/2/2	2/2/2/2	T6.65/T6.65	T6.65/T6.65	4+/3+	4+/3+
11	M/69/L/5	2/3	1+/2	4	4	2/2/3/2	2/2/3/3	T6.65/T6.65	T6.65/T6.65	3+/3+	3+/3+
12	F/72/R/6	2/2	2/2	3	6	2/3/2/3	3/3/3/3	UTF/J4.31	T6.65/J4.31	1+/3+	2+/2+
13	M/59/L/5	1/1	1/0	6	5	6/6/6/4	7/6/6/6	J4.31/J4.31	J4.31/J4.31	3+/2+	3+/2+
14	M/56/L/8	1/1	0/1	5	4	1/1/4/2	1/1/4/2	T6.65/T6.65	D2.83/K4.56	3+/3+	3+/3+
15	M/77/L/8	2/2	2/2	3	5	4/5/5/3	5/5/5/3	UTF/T6.65	T6.65/T6.65	3+/3+	3+/3+
16	F/63/L/13	1/2	1/2	5	4	2/2/3/4	2/2/5/5	T6.65/K4.56	D2.83/D2.83	3+/1+	3+/1+
17	M/71/R/6	1/2	1/2	4	4	3/2/4/4	4/2/4/4	F3.61/J4.31	F3.61/F3.61	2+/3+	2+/2+
18	M/62/R/8	1+/2	1/2	4	5	4/3/4/5	4/3/5/5	D2.83/D2.83	D2.83/D2.83	3+/3+	3+/2+
19	M/78/L/29	3/1	2/1+	4	4	3/3/4/4	3/4/4/4	T6.65/T6.65	J4.31/F3.61	0/0	0/1

Abbreviations: MA, more affected; M, male; F, female; L, left; R, right; FAC, Functional Ambulation Category; A, arm; H, hand; L, leg; F, foot; UTF, unable to feel; S1, 1st sacral spinal segment and L1, 1st lumbar spinal segment.

**Table 2:** Summary of results from within-subject analyses.

Measure	Participants (/19) with Significant Changes after Training	Average Pretest with 95% Confidence Interval and Post-Test Score
Stretch Reflex		
LA SOL	10	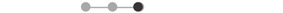
MA SOL *	15	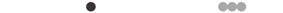
Ratio *	10	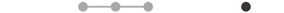
bEMG with arm cycling		
LA iTA	2	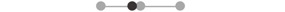
LA cSOL	3	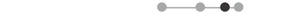
LA cTA	1	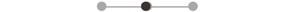
MA iTA	3	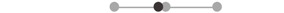
MA cSOL	4	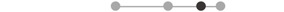
MA cTA	1	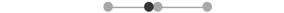
Cutaneous Reflex Modulation Index		
MA SOL	7	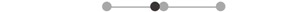
MA TA	8	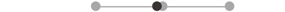
LA SOL	5	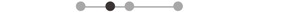
LA TA *	8	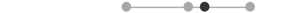
MA FCR *	11	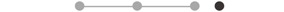
MA PD	8	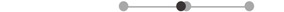
LA FCR	10	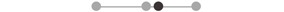
LA PD	10	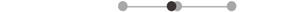
Cutaneous Reflex Modulation Index Ratio		
SOL *	12	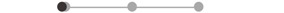
TA *	10	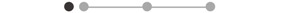
FCR	5	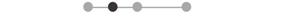
PD	8	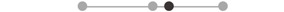
bEMG Modulation Index		
MA SOL	7	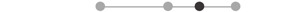
MA TA *	9	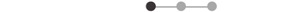
LA SOL	9	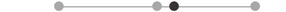
LA TA	10	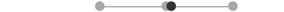
MA FCR *	8	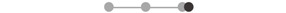
MA PD	7	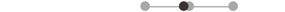
LA FCR	10	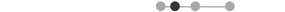
LA PD *	8	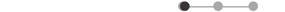
bEMG Modulation Index Ratio		
SOL	4	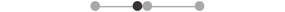
TA *	7	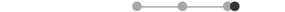
FCR *	10	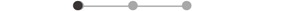
PD	8	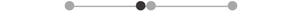

* indicates variables with statistically significant (*p* ≤ 0.05) changes from repeated-measures ANOVA. A graphical representation of the pretest value (denoted with the middle gray circle), the 95% CI (denoted with the outside gray circles), and the post-test value (denoted with the black circle) is given for each variable. Abbreviations: MA, more affected; LA, less affected; i, ipsilateral; c, contralateral SOL, soleus; TA, tibialis anterior; FCR, flexor carpi radialis; PD, posterior deltoid; bEMG, background electromyography.
